# The Distribution Meeting.—£190,000 Awarded

**Published:** 1917-12-22

**Authors:** 


### KING EDWARD'S HOSPITAL FUND FOR LONDON.

### The Distribution Meeting.—£190,000 Awarded.

A meeting of the Governors and General Council of King Edward's Hospital Fund for London, for the purpose of awarding grants to the hospitals, convalescent homes, and consumption sanatoria for the present year, was held at St. James's Palace on December 13, the Speaker of the House of Commons being in the chair.

### Viscount Astor's Donation.

Lord Somerleyton (Hon. Secretary) said that he had the pleasure to announce that the donor of £35,000, who desired to remain anonymous until to-day, was Viscount Astor.

On the motion of the Speaker of the House of Commons it was unanimously resolved that the best thanks of the Council be conveyed to Lord Astor for his very handsome and generous contribution to the Fund.

### A Readily Available Reserve.

Lord Revelstoke (the Honorary Treasurer) said that the total general receipts this year to December 8, less expenses, amounted to £205,781 4s. 9d. This included the donation of £35,000 from Lord Astor to which Lord Somerleyton had just referred. It also included the final instalments of £25,567 10s. 5d. received from Sir Walter Trower on account of the bequest of the late Isabella Countess of Wilton of the residue of her estate. The total residue amounted to no less than £163,000. It was only because of such large contributions as these that the Fund was able to face with equanimity so large a distribution as £190,000. The Finance Committee was making it part of its business to see that the undistributed balance of these gifts was kept in such condition as to be readily available, so that the Council might have at its disposal a reserve force on which to draw in case of necessity.

Sir William Collins, in making the annual statement on behalf of the League of Mercy, said that though the funds collected during the year were not actually paid in to the League until December 15, he thought he could arrive at an approximate figure. Since 1899 the contributions from the League to the Fund had varied from £1,000 to £19,000: during the years 1913, 1914, and 1915 the contribution was £14,000; whilst last year it was raised to £15,000; and the Hon. Treasurer, in view of the amounts at present known, was of opinion that the League would be able to contribute not less than the £15,000 which was handed over last year. Should this prove to be the case, he believed that the total amount which would have been contributed by the League to the Fund would be £260,000. He might add that besides this sum the League gave grants to hospitals outside the County of London, and £25,000 had been distributed in that way, making altogether a total of £285,000 which the League of Mercy had collected and distributed since its inception.

Sir William Church (the Chairman of the Distribution Committee) presented the Report of that Committee (see page 251), and Mr. Frederick M. Fry (Hon Secretary) presented the list of awards (see page 252).

Dr. Edwin Freshfield (the Chairman of the Convalescent Homes Committee) presented the Report of that Committee (see page 253), and Mr. John G. Griffiths (Hon. Secretary) presented the list of awards (see page 254).

### H.M. the King to the Governors.

The Speaker of the House of Commons, in moving the adoption of the Report and Awards, said :—

My Lords and Gentlemen,—Before moving the adoption of the Reports, I have the honour to read the following letter which His Majesty the King, our Patron, has been pleased to address to the Governors through Lord Stamfordham :—

"Buckingham Palace,
"December 10, 1917.

"Dear Sir,—I am commanded by the King to convey to you and through you to the Council of the Fund his congratulations on the distribution of the large sum of £190,000, and his appreciation of the work accomplished during the year by the Governors, the Council, the Committees, Visitors, and other officers of the Fund.

"His Majesty is gratified to learn that so great an increase in the total is largely the result of a most generous gift of £35,000 from a friend whose anonymity has at his own desire been hitherto preserved.

"This considerable addition to the amount for distribution will be of the utmost benefit. The King's many visits to London hospitals during the current year have enabled him to estimate the importance of the services rendered by these institutions to the Navy, the Army, and to men disabled during the war; while the ever-growing demands upon them for adequate ministration to the sufferings of the civil population have equally been brought home to him.

"His Majesty realises also the advance in medical and surgical science as evidenced by the work performed at the hospitals of London. In many cases limbs have been saved, and indeed valuable lives preserved which in the no distant past must have been sacrificed.

"The King is glad to see that the Distribution Committee have paid special attention this year to the accommodation provided for nurses, to whose services every hospital patient would gladly testify. The Reports of the Visitors have shown that the importance of due solicitude for the health and comfort of these workers is realised by the responsible authorities of the hospitals.

"His Majesty is confident that the public will continue to recognise how much has been accomplished by the Fund, and will by their support enable the distribution to be maintained during the difficult times to come.

"Believe me,
"Yours very truly,
"Stamfordham.

"The Presiding Governor,
"King Edward's Hospital Fund for London."

### The Speaker's Address.

It is very gratifying that the Fund should again have been enabled, by the munificence of a very generous donor, to increase its distribution to meet the growing needs of the hospitals in these troublous times. In 1916 it was the bequest of the late Dowager Countess of Wilton that rendered it possible to rise, in one year, from £140,000 to £170,000, without undue risk of a relapse before the need for exceptional efforts was past. This year we are able, through the noble gift of £35,000 from the friend whom we now know to be Lord Astor, to add another £20,000, thus raising the total to £190,000.

### KING EDWARD'S HOSPITAL FUND—(*continued*).

Of course, we do not distribute the whole of such a large gift in one year. That would indeed be rash finance, in a time of long-continued difficulty, the end of which no one can foresee. We consider that the best value for the hospitals is obtained from such gifts if they are used, partly for an immediate increase of the distribution, and partly for its maintenance at or near the increased level over the remaining years of crisis.

### The Backbone of the Annual Subscription.

We could not, of course, hope to do this if we had to rely solely on exceptional contributions of this sort. We have our subscription list, headed by His Majesty the King, our Patron, and including the annual £4,000 from the Drapers' Company and £5,000 from Mr. Walter Morison, himself an active and vigilant member of the Council. We have the annual contribution of the League of Mercy, the result not of spasmodic efforts, but of the continued perseverance and enthusiasm of a well-organised body of workers. And we have a very reliable, though unfortunately not very numerous, list of donors. These together make up a large part, and an essential part, of our regular receipts. Then we have our income from investments, maintained by the ceaseless care of Lord Revelstoke and the Finance Committee. And lastly, we have legacies. These are, of course, very uncertain, and, apart from the exceptional bequest of Lady Wilton, and some receipts from the Lewis Estate, they have dropped from £33,000 last year to £14,000 this year. Thus the gift of £35,000 has saved us from a very real difficulty, which would otherwise have prevented any increase in the distribution.

### Increased Needs to be Met by the Fund.

Now for the application of this unprecedented sum of £190,000. The bulk of it goes in grants to ordinary maintenance, where it is greatly needed to assist the hospitals to bear the strain of high prices and other difficulties. From the figures printed in the Fund's Statistical Report it appears that the ordinary expenditure in 1916 falling on the normal sources of hospital income (that is, not covered by War Office payments for the treatment of wounded soldiers) was £104,000 more than in 1915, and £171,000 more than in 1913. That is the measure of the increased need which this Fund has to do its share in meeting, and no doubt the figures for 1917 will be still larger. Towards this extra £104,000 we contributed in 1916 an extra £28,000; so you will see that there is ample reason why we should again increase our total as far as we safely can.

### The Way to Get Extra Help.

Last year, part of the increased amount given to maintenance was distributed in the form of grants to reduce debt: these grants being given after special inquiry, on the understanding that greater efforts should be made to collect funds from the public. This advice has borne good fruit, and this year you will notice some extra grants, "in recognition and aid of special efforts that have been made to extricate the hospital concerned from serious financial difficulties." On the other hand, there are one or two cases where the hospital is to be informed privately that a special grant to reduce debt would have been made had it not been that the cost of working, being already exceptionally high, had increased out of all proportion to the general rise in cost due to causes common to all hospitals. I hope these three public announcements, of what has often been our private practice, will enlighten the eyes of any hospital managers or others who may be in danger of being misled by the cry occasionally raised, that the way to get extra help from the King's Fund is to run into debt through indolence or extravagance.

Another illustration of the practice of the Fund occurs in the case of Charing Cross Hospital, where the Fund is making an exceptionally large grant this year, to complete the extinction of the mortgage debt. The disappearance of this debt, which only a few years ago was £85,000, is a very notable achievement, and reflects great credit on the present energetic management.

### Nurses' Quarters Specially Inspected.

The Council will notice that the Fund's Visitors were asked, this year, to make a special inspection of the nurses' quarters at all hospitals. This plan of "starring" a particular set of questions in the Visitors' Report for special inquiry in a given year seems to me a very good one. The choice of the nurses' quarters this year was a happy thought, when nurses are harder pressed than ever, and have, even more than usual, the right to expect that their health and comfort shall receive adequate attention from hospital committees. We are glad to see that the reports show that this is the case. Sometimes the buildings in which they are housed are out of date, and yet cannot be replaced during the war. But even here temporary improvements are often possible.

### More Beds in Consumption Sanatoria.

The Convalescent Homes Committee have been able to increase from fifty-three to sixty-two the number of beds in consumption sanatoria placed at the disposal of patients in London hospitals, and also to increase the number of hospitals whose patients can thus be assisted. This provision has been found very useful in the past. Its extension is the result of the increased distribution, and, therefore, counts amongst the benefits which the hospitals owe to the special donation of Lord Astor, our hitherto anonymous donor.

### The Committee on Pensions.

Much progress has been made during the year with the Report on the question of Pensions for Hospital Officers, which a Sub-Committee of the Executive Committee is preparing under the chairmanship of Mr. Whittall. I feel sure that this Report, which contains a great deal of information about Pension Funds in general, will prove a valuable work of reference, not only to hospitals, but to all who have occasion to consider the formation or administration of pension schemes of any kind.

### King George's Fund for Sailors.

Looking outside the Fund for one moment, I should like to be allowed, in the name of the Governors and General Council, to give a word of welcome to the newly established charity which bears, like this Fund, the name of a Royal founder. I mean King George's Fund for Sailors, which will do for marine charities something of the same sort of work as King Edward's Hospital Fund does for hospitals. King George's Fund for Sailors has, of course, no connection whatever with King Edward's Hospital Fund for London, but if in any way our experience, extending now over twenty years, can help the new Fund in its work, we shall all be delighted.

It is with great regret that the Governors record the loss of one of the leading members of the Fund by the death of the Duke of Norfolk, who had served on the

### KING EDWARD'S HOSPITAL FUND—(*continued*).

Council since its formation. The Fund also deplores the loss of the valuable services of three of the visitors: Surgeon-General Sir Benjamin Franklin, Mr. Arthur James, and Mr. David Simson. I should also add that Professor Sir William Osler, who had been a member of the Council since 1908, found himself unable to accept re-appointment this year.

Out of the six members of the office staff, three have at one time or another been on active service; and we regret to say that one, Mr. F. G. Simmons, has recently died of wounds received in the East.

In conclusion, the Governors offer their most sincere thanks to all the members of the Council, members of Committees, Visitors, Honorary Secretaries, and other officers, for their services during another year.

I have now to move the adoption of the Reports.

Sir Arthur Stanley having seconded the motion, the adoption of the Reports was then put and carried unanimously.

On the motion of Mr. Walter Morrison, seconded by Mr. W. J. H. Whittall, it was resolved "that the cheques for the grants should be posted on Wednesday, December 19, except in the case of grants for the reservation of beds in consumption sanatoria during 1918, which should be posted on January 1."

### Arrangements for 1918.

The Speaker of the House of Commons then said:—

My Lords and Gentlemen,—We have now to consider the resolutions delegating powers to the various Committees for 1918, which, if approved here, will be passed at the formal meeting on January 7. The only alteration is in the maximum limit placed upon the amount which may be spent by the Executive Committee. As you know, our expenses are very small, averaging only £1 4s. 3d. in every £100 received. But, like other institutions, we find our costs affected by the war; and the Executive have been compelled to ask that the maximum for ordinary expenses may be put at £4,000 instead of £3,500.

On the motion of Sir Francis H. Champneys, seconded by Sir Horace Marshall, a Resolution was approved delegating powers to the Finance Committee, Distribution Committee, and Convalescent Homes Committee.

On the motion of Sir T. Vezey Strong, seconded by Sir Cooper Perry, a Resolution was approved delegating further powers to the Finance Committee.

On the motion of Lord Knutsford, seconded by Sir John Tweedy, a Resolution was approved delegating to the Distribution Committee the power to draw upon the grants set aside for the amalgamation of throat hospitals.

On the motion of Lord Farquhar, seconded by the President of the Royal College of Physicians (Sir Frederick Taylor), a Resolution was approved delegating powers to the Executive Committee.

Lord Mersey, in proposing a vote of thanks to the Speaker of the House of Commons for presiding, said the loyalty of the Speaker to the King's Fund and its interests was well known.

The Resolution was seconded by the Bishop of London and carried unanimously.

The Speaker having briefly acknowledged the vote of thanks, the proceedings then terminated.

